# The social anatomy of climate change denial in the United States

**DOI:** 10.1038/s41598-023-50591-6

**Published:** 2024-02-14

**Authors:** Dimitrios Gounaridis, Joshua P. Newell

**Affiliations:** School for Environment and Sustainability, University of Michigan, Ann Arbor, MI 48109 USA

**Keywords:** Environmental social sciences, Climate-change policy

## Abstract

Using data from Twitter (now X), this study deploys artificial intelligence (AI) and network analysis to map and profile climate change denialism across the United States. We estimate that 14.8% of Americans do not believe in climate change. This denialism is highest in the central and southern U.S. However, it also persists in clusters within states (e.g., California) where belief in climate change is high. Political affiliation has the strongest correlation, followed by level of education, COVID-19 vaccination rates, carbon intensity of the regional economy, and income. The analysis reveals how a coordinated social media network uses periodic events, such as cold weather and climate conferences, to sow disbelief about climate change and science, in general. Donald Trump was the strongest influencer in this network, followed by conservative media outlets and right-wing activists. As a form of knowledge vulnerability, climate denialism renders communities unprepared to take steps to increase resilience. As with other forms of misinformation, social media companies (e.g., X, Facebook, YouTube, TikTok) should flag accounts that spread falsehoods about climate change and collaborate on targeted educational campaigns.

## Introduction

Climate change denialism persists in the United States, with estimates ranging from 12% to 26% of the U.S. population^1,2^. It is more pronounced in some states and regions^3^. Reasons for this denialism are multifaceted: Political affiliation and ideology, income, education, and exposure to extreme weather events are all important factors^4–6^. Denialism is more prevalent where local economies are highly dependent on fossil fuels^7^, in rural communities, and in populations where mistrust in science is pronounced^8,9^. Social media reaches millions of users, providing a key mechanism for influencers to spread misinformation^10^. The ability of social media to influence and harden attitudes was apparent in the response to COVID-19 vaccines^11^.

Understanding how and why climate change opinion varies geographically and documenting it at an actionable scale is crucial for communication campaigns, outreach, and other interventions^12,13^. Most estimates of the extent and geographic configuration of climate change denialism rely primarily on national surveys, with the Yale Climate Opinion Survey being the only dataset that provides estimates at the state and county levels for the entire U.S.^3^. These survey efforts, however, are time-intensive and expensive and are therefore destined to cover short time spans and, often, limited geographic extent. The Yale Survey combines data from more than 2500 national surveys and uses multinomial regression modeling to downscale estimates to subnational levels. Independent representative surveys conducted in states and metropolitan areas validate the predictions from the Yale Survey models^3^.

Mining social media data (e.g., Facebook, YouTube, and X, formerly Twitter) is a tantalizing alternative to survey-based approaches^14,15^. X is a social media platform with an extensive data repository. By adjusting for the skew toward certain demographic groups in users, data from this platform is useful for estimating public views on an array of topics, such as politics, social issues, and COVID-19 vaccination rates^16,17^. Data from Twitter has also been used in predictive modeling of election outcomes^18^. Account holders can misuse it to oppose scientific knowledge and spread misinformation^19^.

This study used Twitter data (2017–2019) to: (i) estimate the prevalence of climate change denialism at the state and county levels; (ii) identify typical profiles of climate change deniers; (iii) understand how social media promulgates climate change denialism through key influencers; and (iv) determine how world events are leveraged to promulgate attitudes about climate change.

We used a Deep Learning text recognition model to classify 7.4 million geocoded tweets containing keywords related to climate change. Posted by 1.3 million unique users in the U.S., these tweets were collected between September 2017 and May 2019 (see Online Methods S1). We classified these tweets about climate change into ‘for’ (belief) and ‘against’ (denial). Our analysis resulted in a profile of climate change deniers at the county level, provided insight into the networks of social media figures influential in promoting climate change denial, and generated insight into how these influencers use current events to foster this denial.

After confirming the validity of using social media data instead of information collected through surveys to capture public opinion on climate change at policy-relevant geographical scales, we found that denialism clusters in particular regions (and counties) of the country and amongst certain socio-demographic groups. Our analysis reveals how politicians, media figures, and conservative activists promulgated misinformation in the Twittersphere. It maps out how denialists and climate change believers have formed mostly separate Twitter communities, creating echo chambers. Such information provides a basis for developing strategies to counter this knowledge vulnerability and reduce the spread of mis- or disinformation by targeting the communities most at risk of not adopting measaures to increase resilience to the effects of climate change.

## Results

### Where in the U.S. is climate change denial prevalent?

**Figure 1:**
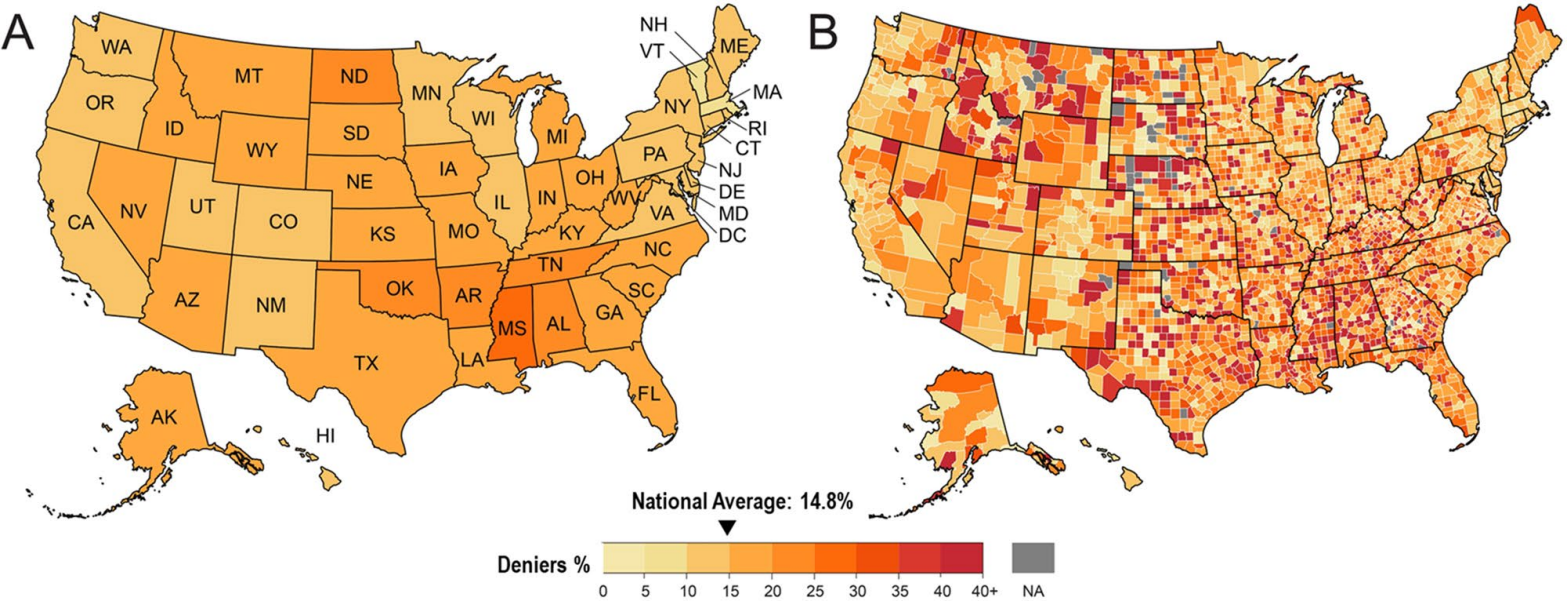
Climate change denialism in the United States, by state (**A**) and county (**B**). Note: Figure created using QGIS 3.30 (https://www.qgis.org/).

Our study found that 14.8% of Americans deny that climate change is real (Fig. 1A), a percentage consistent with previous national studies (Fig. S4). Using geolocation information, we determined that denialism is highest in the Central part of the U.S. and in the South, with more than 20% of the populations of OK, MS, AL, and ND consisting of deniers. Along the West and East Coasts and New England, belief in climate change is highest. However, climate change denial varies substantially within states, often clustering in geographic swaths across multiple counties (Fig. 1B). For example, in Shasta County, California climate change denial is as high as 52%; yet overall less than 12% of the population of California does not believe in climate change. Similarly, the average percentage of deniers is 21% in Texas, but at the county-level this ranges from 13% in Travis County to 67% in Hockley County.

To validate these results, we compared them to the Yale Climate Opinion Surveys at the national, state, and county levels (Fig. S5). The mean absolute difference between the two models was three percentage points (S.D. = 2.7), with the Twitter data yielding a higher percentage of deniers (Fig. S5A). Compared to the Yale Survey, our model showed higher proportions of deniers in Southern states (for example, MS, AL, TN, and TX). However, state-level and county-level percentages of believers and deniers were highly correlated between the two datasets (p < 0.001) (Fig. S5B–E).

### What type of people are climate change deniers?

We performed bivariate correlation analysis with data from multiple publicly available sources (see Online Methods S1) to characterize climate change deniers (Table 1). We evaluated the following characteristics of populations in those regions that were associated with the Twitter profiles for a positive or negative correlation with climate change denial: Political affiliation, race, income, education level, COVID-19 vaccination rates (proxy for belief in science in general), degree of carbon-intensity of the regional economy, degree of urbanization (county-level), and local weather patterns (Table 1). At both the county and state levels, populations with a high percentage of Republican voters had the strongest correlation with climate change deniers. Carbon dependency of the economy was also significantly high at the state level. The strongest negative correlations (at both state and county levels) were level of education and COVID-19 vaccination rates. Integrating these data into a weighted least squares regression model, we defined a profile of a "typical" climate change denier (Table 2). This was the typical profile : Republican, with no college degree and without COVID-19 vaccination living in a region with a high average annual temperature.

**Table 1:** Weighted Pearson correlations, state-level and county-level.

Variables	State level	County level
Political affiliation (Republican)	0.86***	0.63***
Educational level (% of population with a college degree)	− 0.79***	− 0.49***
COVID-19 vaccination rate	− 0.77***	− 0.48***
Carbon intensity of regional economy	0.75***	/
Median household income	− 0.73***	− 0.33***
Urbanization percentage	/	0.30***
Race—Asian	− 0.42*	− 0.32***
Weather—mean temperature (2015-2019)	0.46**	0.25***
Race—White	0.27	0.22***
Weather—National Risk Index	− 0.27	− 0.13***
Race—Black or African American	0.046*	− 0.12***
Weather—temperature anomalies	− 0.13	− 0.02

Note: Total number of tweets per county and per state were used as the universal weights in the model. Significant levels after Bonferroni correction: *Bonferroni correction p < 2.27e^−3^; **Bonferroni correction p < 4.55e^−4^; ***Bonferroni correction p < 4.55e^−5^.

Note: Significant values are in bold.

**Table 2:** Results of the weighted least squares regression model, county level (N = 1960).

Variables	Coefficient	VIF	C.I. 2.5%	C.I. 97.5%
(Intercept)	1384.31		1187.59	1581.02
Political affiliation (Republican)	0.16***	2.33	0.14	0.18
COVID-19 vaccination rate	− 0.09***	1.73	− 0.12	− 0.07
Educational level (% of population with a college degree)	− 0.06***	3.63	− 0.10	− 0.03
Weather—National Risk Index	0.87	1.80	− 0.17	1.90
Weather—Mean temperature (2015–2019)	14.70***	1.16	10.30	19.10
Median household income	0.01	2.57	0.00	0.03
Adjusted R-squared: 0.47	***p < 0.001			

Note: Total number of tweets per county was used as the universal weights in the model. Counties with less than 50 tweets were excluded. Variance Inflation Factor (VIF) < 5 indicates low collinearity of the variables used in the model.

*C.I.* confidence intervals of regression coefficients.

Note: Significant values are in bold.

**Figure 2:**
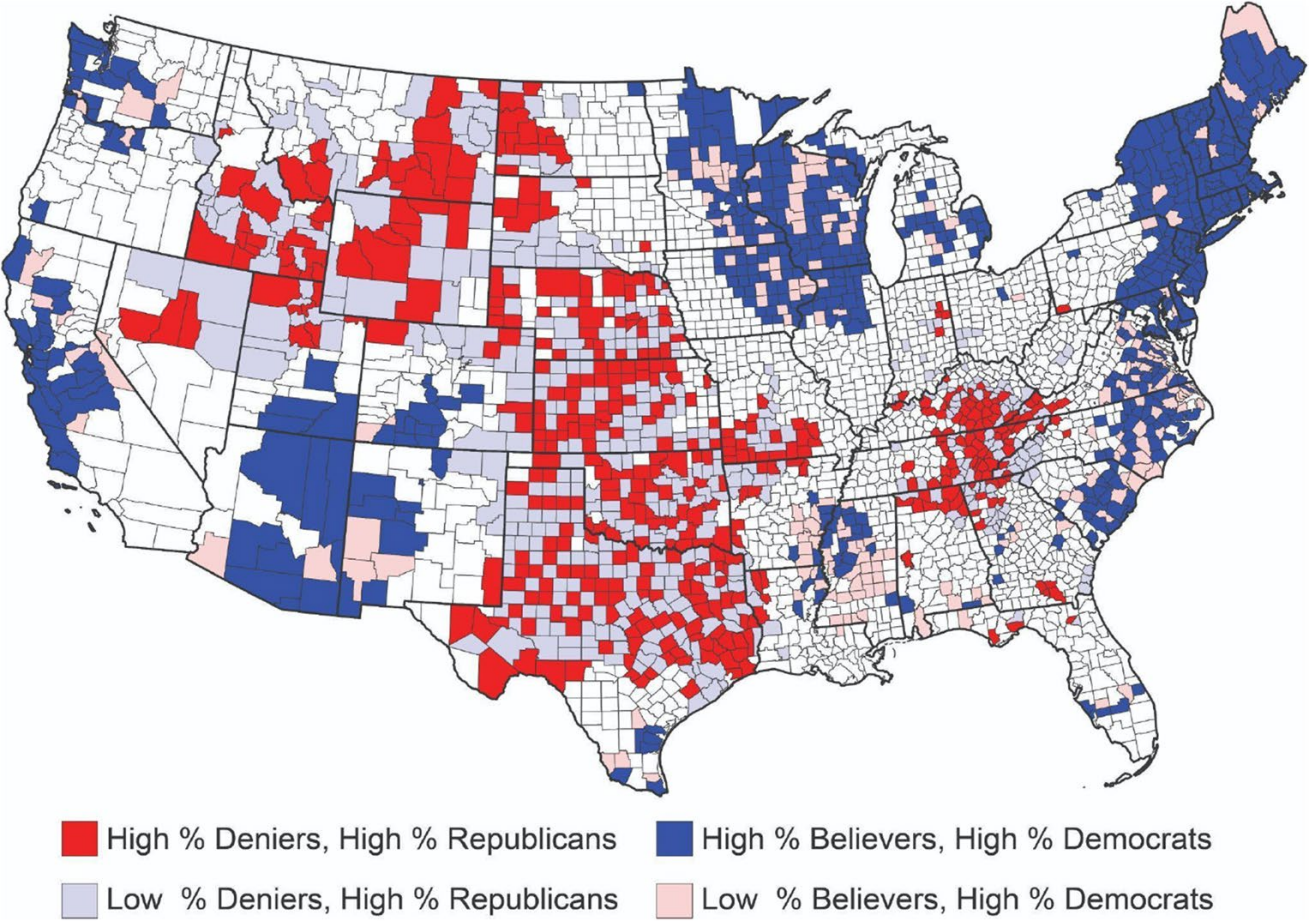
Clusters of spatial association between climate change denial and belief in relation to political affiliation. Notes: Figure created using QGIS 3.30 (https://www.qgis.org/). Spatial clustering analysis performed using Geoda 1.22 (https://github.com/GeoDaCenter/geoda/).

To gain additional insight into the geographical relationship between denialism and political affiliation at the county level, we used the bivariate LISA (Local Indicators of Spatial Association) model^20^ to identify which counties with high rates of denialism or belief were spatially associated with high rates of Republican or Democratic voters. Clusters of deniers that coincided with high rates of Republican voters were spatially contiguous and covered large swaths of the interior West (Idaho, Montana, Wyoming), Central (Nebraska, Kansas, Oklahoma, Texas), and Appalachian regions (West Virginia, Tennessee) of the U.S. (Fig. 2). These findings are consistent with our regression modeling and bivariate correlations: these regions tend to have high rates of carbon dependency of the economy, low COVID-19 vaccination rates, and large rural populations. Conversely, clusters of believers and high rates of Democratic voters were most prevalent along the Pacific Coast (California, Washington), the New England Region, the Great Lakes, and the Southwest (Arizona), as well as in regions near metropolitan areas and technological hubs.

### Who are climate change influencers in the Twittersphere?

**Figure 3:**
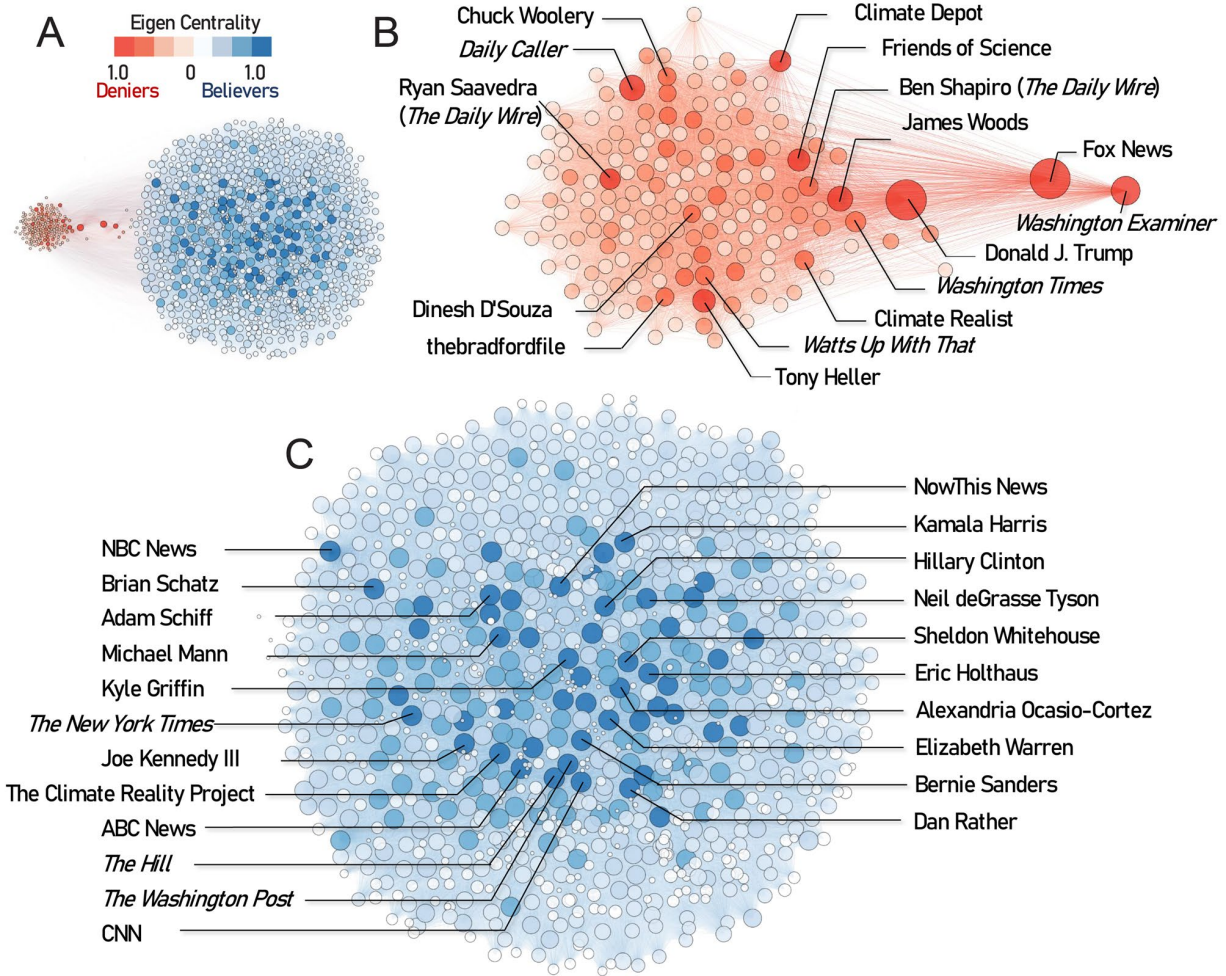
Influencers detected in climate change co-retweeted networks. (**A**) Co-retweeted networks formed by the 1200 most retweeted users in the U.S. The nodes represent unique accounts; the edges represent co-retweeted relationships. The size of nodes and the shade of the node color are proportional to their influence, as measured by eigenvector centrality scores. The high density of edges within the communities makes many individual edges not displayable. The top influencers in the community of climate change deniers (**B**) and believers (**C**) are labeled with the usernames. In panels (**B**) and (**C**), edges-to-users in the other community are not displayed.

To delineate how polarized opinion forms in the Twittersphere, we constructed Twitter networks (based on the 1200 most retweeted users in the sample), analyzed how users interact, and identified key influencers (Fig. 3). To identify closely linked users assumed to share similar views, we evaluated co-retweeting, in which a single user retweets tweets from two or more other users^21^. Two distinct communities emerged, a denier community and a believer community (Fig. 3A). The community of climate change believers (blue nodes) is larger, with 1029 users and ~ 224,000 co-retweets, giving it a broader reach and influence on Twitter than the denier community (red nodes), which has 171 users and ~ 15,000 co-retweets. The proportion of deniers among the top 1200 influential users (14.3%) aligned with the national percentage of climate change deniers identified in our model (14.8%).

Both believers and deniers mostly shared information and interacted within their own community. Users from the two communities were rarely co-retweeted, as illustrated by the distance between the cluster of nodes for each community and the low number of edges connecting the two communities. Among ~ 230,000 co-retweets, only 4083 (< 0.02%) were between users having opposite views on climate change. This low percentage of co-retweets of contrasting views highlights an *echo-chamber effect*. We found that a few nodes bridge the gap between the two communities, notably conservative news outlets such as *Fox News* and the *Washington Examiner*.

To identify the most influential users, we calculated the eigenvector centrality value per Twitter user. A high score means that a user was co-retweeted with many other users who also had high scores. Among climate change deniers, former U.S. President Donald Trump had the biggest influence (Fig. 3B). Three groups of influential deniers were heavily co-retweeted with President Trump: (i) conservative media outlets that regularly broadcast contrarian views on climate change, including alt-right news and blogs such as *The Daily Wire*, *Daily Caller*, *Breitbart* and *thebradfordfile*; (ii) mis/disinformation websites that publish misleading and false claims about climate change, including *TownHall Media* and the *Climate Depot*; and (iii) right-wing producers, political commentators, and activists. Collectively, in concert with former President Trump and close colleagues, these three groups formed an organized and coordinated social media network, enabling climate change denialism to amplify and expand.

In contrast, the larger blue community is more diffuse. Politicians dominated the most influential users (Fig. 3C). Of the top 30 influential believers, 15 accounts belong to members of the Democratic Party, such as Alexandria Ocasio-Cortez, Bernie Sanders, and Kamala Harris (Table S1). Eight of the top 30 nodes were popular media outlets, or websites, such as CNN, NBC, ABC, *The Hill, The Washington Post, The New York Times*. Other influential nodes included popular science communicators and entertainers advocating scientific consensus.

### How does climate change-related tweeting and topic use vary over time?

To investigate the dynamics of tweeting activity for both communities and to understand how each perceived and responded to real-world events, we performed topic modeling and time series analysis of tweet volume. This analysis revealed how each group reacted selectively and opportunistically to the 17 events that occurred during the period of data collection (November 2017–May 2019).

**Figure 4:**
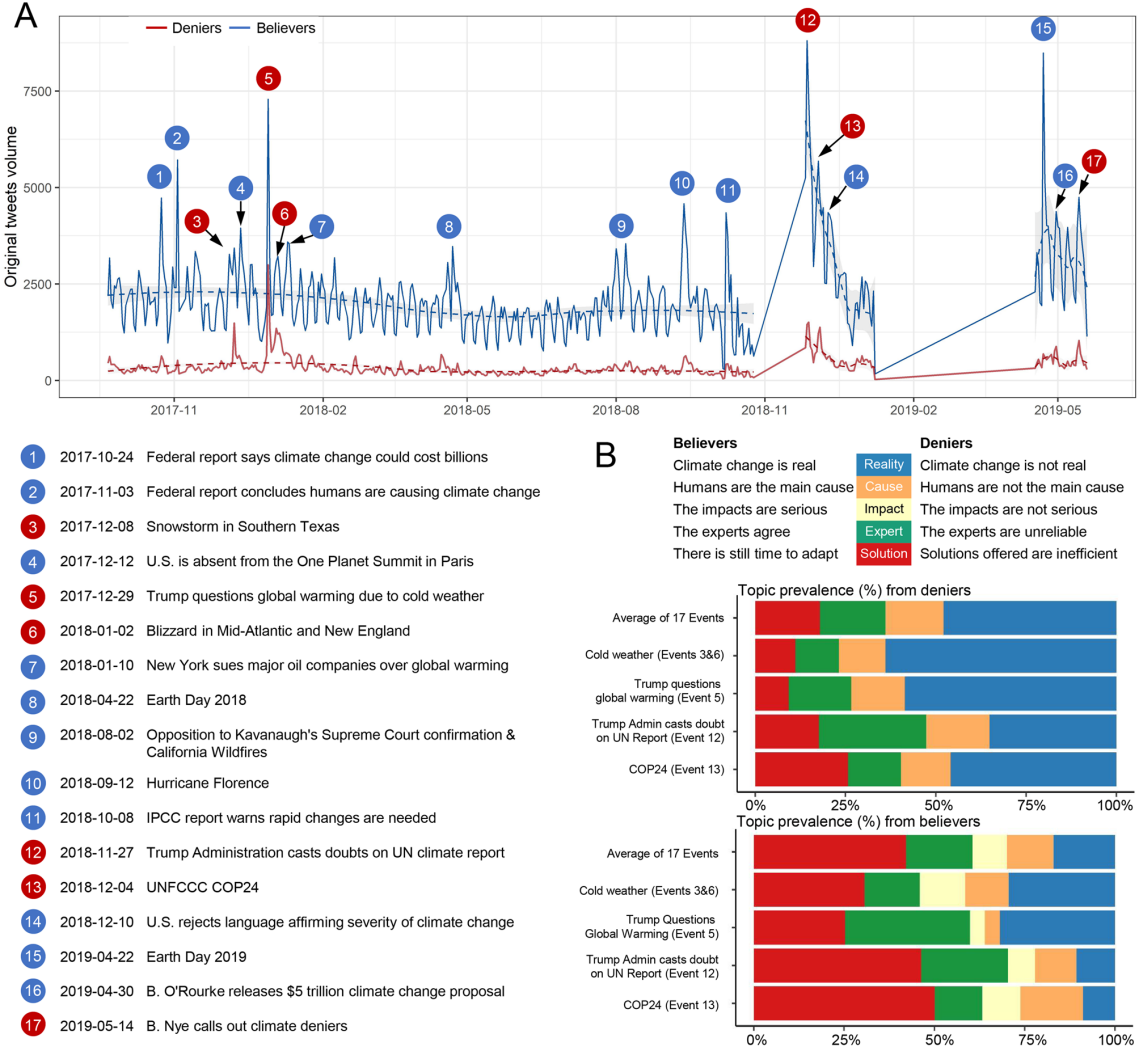
Events that drive tweet volume among deniers and believers and topic prevalence for typical events. (**A**) Original tweet volume per day and locally weighted regression lines are plotted over time for both climate change deniers and believers. Events that sparked online discussions are labeled alongside tweet volume spikes numerically and detailed in the lower left. Red bubbles denote the events that a large group of deniers were actively involved with (> 1000 original tweets). The gap in November 2018 and between January and April 2019 was due to discontinued data collection. (**B**) Topic prevalence for typical major events^22^: Events 3 and 6 represent extreme cold weather events; Event 5 represents top denier influencer Donald Trump tweeting about cold weather and doubts about global warming; Event 12 represents the Trump Administration publicly refuting the validity of the IPCC 2018 report (https://doi.org/10.1017/9781009157940); and Event 13, a UN climate change conference (COP24), represents an event that engaged both deniers and believers.

Consistent with the larger size of the believer community, this community had a consistent pattern of climate change tweet activity throughout the sampling period (Fig. 4A). In contrast, the denier community had lower activity overall. However, both communities had periods of high activity with spikes that exceeded the average pattern. The number of these high spikes was lower for the denier community. By manually identifying events that potentially triggered these large spikes, we found that deniers and believers do not always respond to the same events. Only six events triggered higher than average tweet volume by the denier community (Table S3): three were related to extreme cold weather events, two were related to United Nations activities about climate change —a report by the Intergovernmental Panel on Climate Change (IPCC) and an annual meeting of the United Nations Framework Convention on Climate Change (COP24), and the last was an attack on climate change deniers by Bill Nye in an HBO broadcast. Intriguingly, two of the highest spikes by the believer community occurred with events associated with President Trump that sparked high activity in the denier community, suggesting that these communities tried to influence or counter each other.

To gain further insight into whether the groups attempted to counter each other, we classified tweets of believers and deniers for these 17 events based on the five climate change narratives (see Fig. 4B) proposed by Cook^22^. Overall, the major narrative in the believer community was “There is still time to adapt," representing 42% of the total tweets). In contrast, deniers focused tweeting activity on the message “Climate change is not real,” as indicated by 48% of the tweets falling into this category.

Although weather events were associated with spikes in tweets from both communities, events viewed as abnormal weather caused by climate change [the California Wildfires (Event 9) and Hurricane Florence (Event 10) triggered a high volume of tweets among believers and events viewed as colder-than-expected weather [a snowstorm in Texas (Event 3) and a blizzard in the Mid-Atlantic and New England regions (Event 6) triggered a surge in tweets amongst deniers. Both colder-than-expected weather events provided an opportunity for the deniers to espouse that climate change is not real (64% of the total tweets for both events), to delegitimize scientific consensus (12% of the total) and to reaffirm the claim that the changing climate is a normal geologic process and to foment doubt that human activities are a source of this change (13% of the total).

Consistent with an attempt to counter each other’s messages, the December 2017 tweet by Trump casting doubt on global warming due to a blizzard (Event 5) triggered the believer community to issue tweets emphasizing that climate change is unequivocal (32% of the total) and that there is clear scientific consensus (35% of the total). A common refrain among deniers was that climate change is a conspiracy theory or hoax (59% of the total) and a shadowy attempt to dupe the public into bearing the costs of decarbonization, while generating enormous wealth for Blue ‘elites’ (9% of the total). These tweets were heavily re-tweeted by conservative media (e.g., Daily Caller), right-wing activists (e.g., Chuck Woolery), and mis/disinformation sites (e.g., Watts Up With That?) (Table S1).

Conflicting messages were also common in response to COP24, consistent with an attempt to influence opinion. Believers overwhelmingly advocated for timely collective action or promoted campaigns showcasing impacts of and solutions to climate change (50% of the total). Deniers focused on conspiracy theories (climate change is not real, 46%) or a Democratic party agenda filled with impractical solutions (26%).

## Study limitations

Our modeling approach has several limitations. First, X (formerly Twitter) has limited the number of posts a user can read in a day, ostensibly to prevent ‘data scraping’ practices from unauthorized users. If this policy persists, then it will hamper research that relies on social media data to assess online beliefs and sentiment. This has implications in terms of the replicability of our modeling to other countries, time frames, and topics^23–25^. A second limitation of our modeling is that uncertainty is higher in areas with low population densities (e.g., rural areas) due to small sample sizes^26^. This is a well-known limitation of using social media to assess consensus and is even more pronounced in countries where use of social media is limited. To minimize this effect in our study, we normalized input data by county population and employed a weighted approach using the total count of tweets as weights both for the calculation of bivariate relationships and for the regression models (see “Methods”). Third, our classification scheme labeled tweets as either believing or denying climate change. National surveys indicate a percentage of the population (5–15%) who remain neutral or may not have a particular opinion on the topic^27^. In our binary classification, we used those climate change-related keywords that indicated a clear position (for or against) on the issue. As a portion of our sample uses sarcastic or ambivalent language that is difficult for the model to distinguish, classifying these tweets can be challenging. To address this, we calculated confidence for each prediction (see “Methods”) to filter out those with low confidence (CI < 0.75).

## Discussion

Using data from Twitter (now X), we used AI techniques and network analysis to delineate a comprehensive social anatomy of climate change denialism in the U.S., at the state and county levels. We identified geographic clusters of climate change denial in Republican counties, especially rural ones, and among residents who do not have a college education. This provides critical knowledge for identifying segments of the population that would benefit from targeted efforts to expand awareness of the risks associated with climate change and strategies to increase local resilience.

The strong correlation between denialism and low COVID-19 vaccination rates indicated a broad skepticism of science generally amongst climate change deniers, which corresponds to resistance to science-based public policies such as shelter-in-place COVID-19 mandates^28^ or mask usage^29^. This finding indicates that communities with a high prevalence of deniers are at risk for discounting other science-based health or safety recommendations. According to the theory of identity-protective cognition, people tend to selectively credit or discredit evidence in patterns that reflect beliefs that predominate in their group^30,31^. This theory helps explain why those who vote Republican are more likely to believe tweets from former President Trump regarding climate change than from other sources; it is identity affirming.

Classifying tweets based on the Cook’s five categories^22^ enables identification of commonly deployed rhetorical strategies to promote climate misinformation, and in science denialism more broadly^32^. In our 7.3 million tweet sample, these techniques included *fake experts,* who have possessed little to no expertise about the underlying science but nonetheless conveyed messages that cast doubt. They serve as a *credible messenger* in which someone shares the same moral values and uses language consistent with existing beliefs^12,33^. One such example is the tweet by the Trump Administration casting doubts on the IPCC 2018 Climate Report, which was retweeted heavily by supporters. Then there are *logical fallacies*, such as a tweet by Trump questioning global warming because of an unusual cold weather event that went viral^34^. Other common strategies include *impossible expectations* as well as *cherry picking* to attack climate change science and scientists.

Combating misinformation requires effective refutation strategies^35^. Deploying such strategies on social media sites such as X, however, is challenging as denier and believer communities are isolated from each other, leading to echo chambers^19^. Only 0.02% of the co-retweets about climate change were between users having opposing views. Consequently, this leads one to conclude that believers have limited ability to reach deniers through the social media platform. One strategy is to label denialism tweets as misinformation. However, some evidence suggests that this can strengthen opposition rather than change attitudes^36^. Another option is to flag accounts that disseminate misinformation or dangerous information. For example, then-Twitter banned Donald Trump (his account has since been restored) from using the site because of tweets maintaining election fraud and supporting the January 6 capital riots^37^. Twitter also banned accounts for spreading COVID-19 misinformation and calling for violence against media^38^. As with COVID-19, climate change is a humanitarian crisis that will affect millions, albeit at a more elongated temporal scale. Based on current policy, X (formerly Twitter) does not appear to be imposing account bans or suspensions for promoting climate change denialism. This, and related policy changes associated with new ownership of the social media platform, may make it even more susceptible to the spread of misinformation.

Communities face increasing risks related to climate change, such as flooding, wildfire, heat stress, and sea-level rise. The scientific community has already identified especially vulnerable communities and regions^39^. Climate change denialism is also a risk, in the form of knowledge vulnerability. Those who discount climate change as a natural rather than human-induced process tend to underestimate their current (and future) risk to it. This renders them less likely to take necessary steps to mitigate and adapt to climate change.

## Methods

### Opinion data

As primary data, we used an open access dataset created by George Washington University that is available from the GWU Libraries Dataverse^40^. This dataset was created using the Twitter Stream API and contains ~ 40 million tweets related to climate change and global warming. It covers a two-year period from September 2017 to May 2019. We initially retrieved ~ 27.3 million raw tweets based on tweet IDs. The ~ 30% loss of tweets was due to deleted or inactive accounts since 2019.

To extract tweets located in the U.S., we developed a rule based on the geo-attributes in the raw data. We extracted the self-reported location information in an account profile. A large proportion of users (> 73%) provided the location information in our dataset. To standardize the addresses and improve the geocoding process, we first transformed all the user locations to lower case and removed the URL links, emojis, punctuation marks, and other non-ASCII characters. Next, we extracted all the unique user locations (~ 640,000 “clean” addresses) and standardized all the U.S. state and city abbreviations. As a final step, we manually inspected and removed national level and obviously fake user locations.

After the preprocessing, we used the Nominatim API server to geocode user locations based on the OpenStreetMap database^41^. We removed locations outside the U.S., and classified addresses within the U.S. into two levels: (1) county level with tweets from users reporting their local address, city, or county; (2) state level with tweets from users reporting only the state. In the state-level tweets, we also added the aggregated county-level tweets. We then rejoined these unique U.S. addresses and the corresponding geographical coordinates to the original datasets by spatial level. The geocoding yielded ~ 1.3 million unique users and ~ 5.2 million county-level tweets and ~ 7.4 million state-level tweets, from which ~ 2.2 million tweets had state-level only information. To reduce the incidence of non-human accounts in our sample (i.e. tweet bots), we removed users who tweeted more than 20 times a day. Figures S1 and S2 presents the data spatial distribution and representativeness analysis.

### Tweet classification

To identify climate change opinions on Twitter, we built a tweet classifier based on the Transformer, a deep learning model in the field of natural language processing^42^. We parameterized the model to classify tweets as either believing in the existence of climate change (predicted as ‘for’) or denying that climate change is real (predicted as ‘against’). Instead of training a model de novo, the Transformer uses language models pre-trained on large text corpora in an unsupervised manner and then uses user-labeled training samples to fine-tune the model for specific natural language tasks. Our classifier was built upon OpenAI GPT-2, a large transformer-based language model pre-trained on a database of ~ 8 million web pages^43^. Previous studies found that the GPT-2 model performs well in classifying short text from social media^44^.

We built a training dataset of manually labeled tweets to fine tune the pre-trained GPT-2 model. Labeled samples were randomly extracted only from the 1.4 million original tweets, excluding retweets and quotes. Each tweet was reviewed independently by two members of the research team and labeled as either ‘against’ or ‘for’ climate change. In rare occasions where a tweet’s message was ambiguous and there was disagreement between the two members of the research team, the tweet was excluded from the model's training data due to its potential to introduce noise to the model.

We labeled training tweets as ‘for’ or ‘against’ climate change if they had one of the following viewpoints listed in Table 3. This labeling resulted in a balanced sample of 6,500 tweets (3300 ‘for’ tweets and 3200 ‘against’ tweets) that we used as a training set for the model. Tweets with ambiguous messages, sarcastic language or tweets that were irrelevant to climate change were discarded from the training dataset.

**Table 3:** Classification of tweets used for training the model as ‘for’ or ‘against’ climate change.

‘For’ (belief): N = 3300 tweets	‘Against’ (denial): N = 3200 tweets
*Climate change concern:* The user believes climate change is real and worries about its negative consequences *Advocate for action:* The user calls for collective actions and supports adaptation and mitigation policies *Scientific consensus:* The user advocates for the scientific evidence on climate change and recognizes the role of greenhouse gas emissions caused by human activities	*Trend denialism:* The user shows disbelief that the Earth is warming and climate change is real *Attribution denialism:* The user believes climate change is happening, but it is a natural, unpreventable process and anthropogenic greenhouse gases are not the dominant driver *Impact denialism:* The user believes climate change will not have significant negative impacts on the environment and humanity *Evidence denialism:* The user doubts there is trustworthy scientific consensus on climate change

Our model was built upon the *Huggingface* Transformers^45^ Library and implemented in *PyTorch*^46^. To increase the model’s predictive accuracy, we fine-tuned the parameters that resulted in an optimum learning rate at 1e−5, with dropouts at 0.1. Tweets with sarcastic, ambiguous or irrelevant messages were evaluated with the model, but the predictions based on these tweets tended to be invalid or random. To overcome this limitation, we used the *Softmax* function embodied in *PyTorch*, which calculated the prediction confidence for every individual tweet. Based on this score, we removed predictions with low confidence (CI < 0.75). The final classification was performed on the complete set of 7.4 million tweets from the collection period. We then aggregated tweets at the county and state levels and calculated percentages of ‘against’ tweets and ‘for’ tweets as proxies of deniers and believers.

To evaluate the model’s performance, we performed a series of validation tests. We manually labeled an independent validation dataset to test model accuracy. To ensure the validation dataset was balanced across the two categories and was spatially representative, we randomly extracted 30 unique original tweets from each state. Our fine-tuned model achieved an overall accuracy of 0.91 and F1 score of 0.90 (Fig. S3). Our model predictions were compared with national estimates of climate change opinion based on representative surveys, showing that our model provided a percentage for U.S. climate change deniers within the range of those determined from the surveys (Fig. S4). To validate our results at the sub-national level, we used the Yale Climate Opinion Surveys. The Yale Surveys use a downscaling statistical model based on national survey data and are the only surveys that provide climate change opinion estimates at the state and county levels. We compared these data with our model results at both state and county levels by calculating the Pearson correlation coefficient. To normalize the data, we weighted the variables per population of each state and county (US Census 2018).

### Correlation analysis

To examine what drives climate change opinion, we performed a series of correlation analyses. Studies have shown that climate change opinion is strongly correlated with political affiliation which is considered a major driver^47,48^. In addition to political ideology, studies have shown evidence that the socio-demographic profile also plays a role in climate change opinion as does the local microclimate^49,50^, and personal experience with extreme weather events, although the evidence is mixed and at times ambivalent^51–53^. Informed by this literature, therefore, we examined variables that have been reported to influence climate change opinion: political affiliation, COVID vaccination rate (proxy for belief in science in general), urbanization rate, education, income, race, carbon intensity of economy, natural hazard risk, and temperature anomalies.

We used the percentage of ‘against’ and ‘for’ tweets to reflect the prevalence of deniers and believers across the U.S. at the county and state levels. For political affiliation, we acquired 20 years (2000–2020) of county-level U.S. Presidential election returns from the MIT Election Data and Science Lab (https://electionlab.mit.edu/data). We calculated the average percentage of Democrats and Republicans per state and county, weighted by the county population. For science skepticism, we used the county-level COVID-19 vaccination rates as a proxy, using data from the CDC (https://www.cdc.gov/coronavirus/2019-ncov/vaccines/distributing/reporting-counties.html). For educational attainment, race, and income, we used data from the US Census Bureau's 2020 American Community Survey, which provides estimates of average characteristics from 2016 through 2020 at the state and county levels. Specifically, we used the number of people who have at least a Bachelor's college degree, number of people per race, and the median household income. For county-level natural hazard risk, we used the National Risk Index developed by FEMA (https://www.fema.gov/flood-maps/products-tools/national-risk-index). An overall risk score was calculated for each county, measuring the expected annual loss due to 18 types of natural hazards. For temperature anomalies, we acquired historic 30-year annual mean temperature (1981–2010) and the mean for recent years (2015–2019) from the PRISM climate group (https://prism.oregonstate.edu/). County-level temperature anomalies were then obtained by calculating the standard deviation between annual mean temperature of recent years and the 30-year averages. To investigate the association between state-level carbon dependency of economy and climate change opinion, we used energy-related carbon emissions per gross domestic product (GDP) for each state from the Energy Information Administration (https://www.eia.gov/environment/emissions/state/). The unit of carbon intensity is the metric tons of energy-related carbon dioxide per million dollars of GDP. A six-level urban–rural classification at the county level was from the National Center for Health Statistics data systems (https://www.cdc.gov/nchs/data_access/urban_rural.htm).

To account for variations in population across counties and states, we normalized all data expressed as counts. We adjusted the total county population as: Population_Adj_ = Total population/10,000. Then, we normalized each variable by population by dividing the counts of people for each variable by the adjusted population: Normalized Variable = Variable count/Population_Adj_. Based on the normalized data, we calculated bivariate weighted Pearson correlations between climate change opinion and each of these variables using the total count of tweets per county as the weight. The same data were used as predictors for the regression model. We used the weighted ordinary least squares for the total count of tweets per county as the universal weight.

To identify spatial clusters of climate change denialism or belief at the county level in relation to political affiliation (Republican or Democrat), we applied the bivariate Local Indicators of Spatial Association (LISA)^20^. We applied the second order Queen contiguity weights at the county level and ran the models with 999 permutations and significance at p < 0.05. This approach was executed in the open-source software *Geoda*^54^.

### Co-retweeted network analysis

We constructed a co-retweeted network to delineate interactions and identify the most influential Twitter users from both sides. Co-retweeting is defined as the act of a single user retweeting two or more other users. We used these events to create undirected weighted edges between the co-retweeted accounts. The more users retweet two other users, the more weight the edge gains. Accordingly, we assumed that the more co-retweets two accounts receive, the more likely their views are related. The co-retweeted network represents engaged communities with similar opinions.

To construct the co-retweeted network, we first calculated the total sum of retweets as a measure of overall influence for each user account in our 7.2 million tweets dataset. We selected the 1200 most retweeted accounts for further processing, along with all the users who have retweeted them. We then constructed the retweet matrix *A* where the rows represent the 1200 top accounts, and the columns represent the rest of user accounts. Elements in matrix *A* are binary: A value of 1 means that the public account has retweeted the corresponding top influential account and 0 means the public account has not retweeted the top influential account. We then multiplied matrix *A* with its transposed matrix *A*^*T*^ and transformed it into the co-retweeted square matrix *B*. Matrix *B* has 1200 rows and columns that represent the influential accounts. The upper and lower diagonal cells of matrix *B* contain the total number of times that two influential accounts are co-retweeted. We exported all the unique pairs of influential accounts and their co-retweets as the edge table for further network analysis.

Our co-retweeted network was visualized in *Gephi*, using the Force Atlas algorithm^55^, which clusters nodes based on their connections. The distance between two nodes was weighted by the number of co-retweets. We then applied the Louvain community-detection algorithm^56^ and separated the nodes as two communities based on modularity scores. To detect opinion leaders in each community, we calculated the Eigen centrality values for each node based on the *igraph* package in R^57^. The number of co-retweets for each node was set as the weight. To facilitate visualization, we extracted the top 30 influencers from each community (Table S1 for deniers and Table S2 for believers). The eigenvalues are scaled to a maximum score of one.

### Time-series analysis and topic modeling

To examine the dynamics of tweeting activity regarding climate change, we analyzed the tweet volume of both deniers and believers during September 2017 to May 2019 and identified 17 major climate change-related events that may spark controversy online. To identify real-world events, we first applied the z-score algorithm to detect spikes in tweets volume of deniers and believers separately, along with the daily time series. The algorithm detected peaks when the value of a new data-point is three standard deviations away from the moving mean of 5 days of observations. The algorithm identified nine peaks in deniers’ tweets and 16 peaks in believers’ tweets. We then applied the tidytext package in R to segment each day’s Twitter texts into separate words and calculate the term frequency of each word. Next, we reviewed these keywords and manually assigned each peak to one real-world event alone. Comparing independent coding results of the three coders, we got a Cohen's kappa score of 0.90, indicating good interrater reliability. Divergences were resolved following discussions among the coders. Table S3 presents the dates with spikes in tweet volume, the most prevalent keywords, and associated real-world events.

To delineate the major climate change-related topics discussed, to understand how the prevalence of each topic evolved over time, and to explore how each group perceived the event, we employed the Latent Dirichlet Allocation (LDA) algorithm^58^ to automatically extract the main topics. We specified the number of topics before training the model. We devised a five-category classification scheme following Cook’s^22^ categories of misinformation: (a) climate change is/is not real; (b) humans are/are not the main cause; (c) the impacts are/are not serious; (d) the experts agree/are unreliable; (e) there is still time to adapt/solutions offered are inefficient.

The model was implemented in Python’s Gensim package along with the Java-based package Mallet to accelerate data processing^59^. We ran topic modeling separately for tweets classified as from ‘believers’ or ‘deniers.’ We preprocessed the original ~ 7.2 million tweets, keeping original tweets and excluding retweets with the same text. We removed all the @mentions, hashtags, punctuation marks, and changed all characters to lower case. From keywords, we removed “climate change” and “global warming” because these words occurred too frequently and would dominate as distinct topics. After this pre-processing, we tokenized every tweet and created bigrams and trigrams because some words often occurred together as phrases. We reduced words to their common word stem and dropped duplicates to ensure the text corpora analyzed by the model was clean and distinct.

### Supplementary Information

Supplementary Information.

## Data Availability

Primary Twitter data and ancillary data were obtained from publicly available sources (see “Methods”). Secondary data that support the findings of this study are available from the corresponding author upon reasonable request.

## Code availability

Code generated to perform this analysis is available at https://zenodo.org/record/8017765.
