# Supplementary material for: The social anatomy of climate change denial in the United States
Source: Sci Rep. 2024 Feb 14;14:2097. doi: 10.1038/s41598-023-50591-6 (PMC10866916; doi:10.1038/s41598-023-50591-6)
Supplement: Supplementary file 1 — Supplementary Information. [file 41598_2023_50591_MOESM1_ESM.docx]

*Supplementary Material for*

The Social Anatomy of Climate Change Denial in the United States

**This file includes:**

Appendix (1): Data Preparation; Appendix (2): Model Validation; Appendix (3): Additional Results; Figures S1 to S5; Tables S1 to S4; SI References

**Appendix (1): Data Preparation**

*Data collection and preparation*

Our study utilized an open access dataset built by researchers from George Washington University who collected ~ 40 million climate change related tweets, via the Twitter Stream API.[^1^](#_heading=h.2et92p0) The dataset contains tweets from around the world posted in a two-year period, from Sept 2017 to May 2019.

We used Hydrator, a desktop application,^[2](#_heading=h.tyjcwt)^ to retrieve raw twitter data based on tweet IDs. In Nov 2020, we successfully retrieved ~27.3 million raw tweets. The following keywords were set as the filters, which contain popular hashtags from both climate change believers and deniers.

#climatechange, #climatechangeisreal, #actonclimate, #globalwarming, #climatechangehoax, #climatedeniers, #climatechangeisfalse, #globalwarminghoax, #climatechangenotreal, climate change, global warming, climate hoax

We then extracted the necessary attributes both for each tweet and the user who posted the tweet. Tweet attributes include the full text, author ID, time of creation, geo location, tweet type (original tweet, retweet, quote, reply), and cumulative number of retweets and likes. User attributes include the username, self-defined user location, and number of followers.

*Spatial distribution of tweets*

After cleaning the self-defined addresses in user profiles (see methods), we used the Nominatim API to geocode user locations.[^3^](#_heading=h.3dy6vkm) **Fig. S1** shows the tweet volume per unique address across the US, where pixel shading is associated with the logged volume of tweets. As expected there is notable spatial variation in tweets, with most tweets coming from users in urban areas and along the coasts. By calculating tweets volume per county, we find over 50% of counties have more than 100 tweets and over 75% of counties have at least 30 tweets.


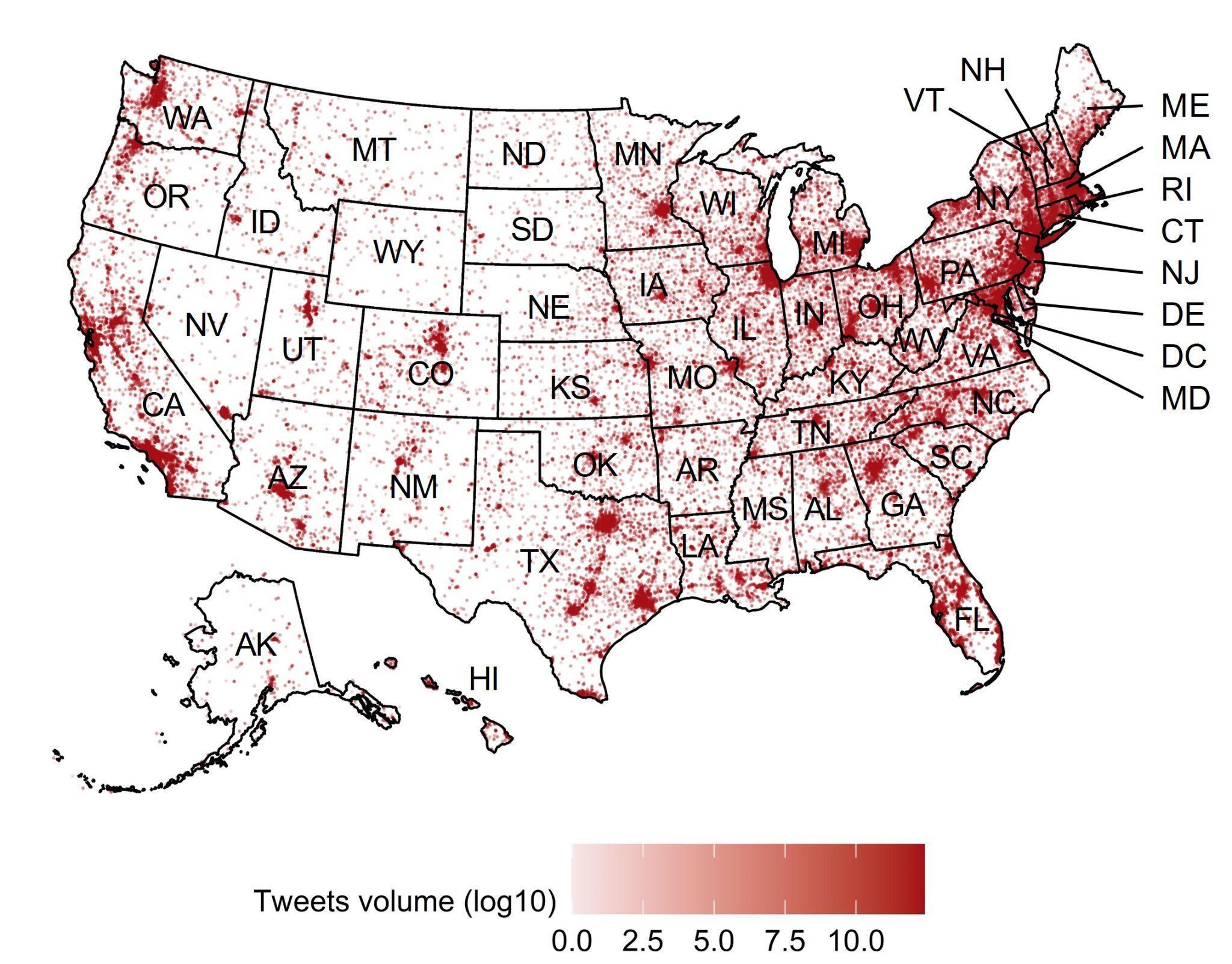


**Fig. S1.** Tweet volume per unique point address used in our analyses. The map was generated by R statistical computing software version 4.3.1 (https://cran.r-project.org/).

*Data representativeness*

To test the representativeness of our dataset, we calculated the total number of users and tweets at the county and state levels and its correlation with the population of the respective jurisdictional level. As shown in **Fig. S2a**, the total number of tweets highly correlates with the population at the state level (R = 0.89, p-value < 0.001). One exception is the District of Columbia, where tweets per capita is significantly higher than that of the national average probably because of its disproportionate number of politicians and environmental organizations located in the city. **Fig. S2b** depicts the correlation of tweets volume by county (R = 0.72, p-value < 0.001).


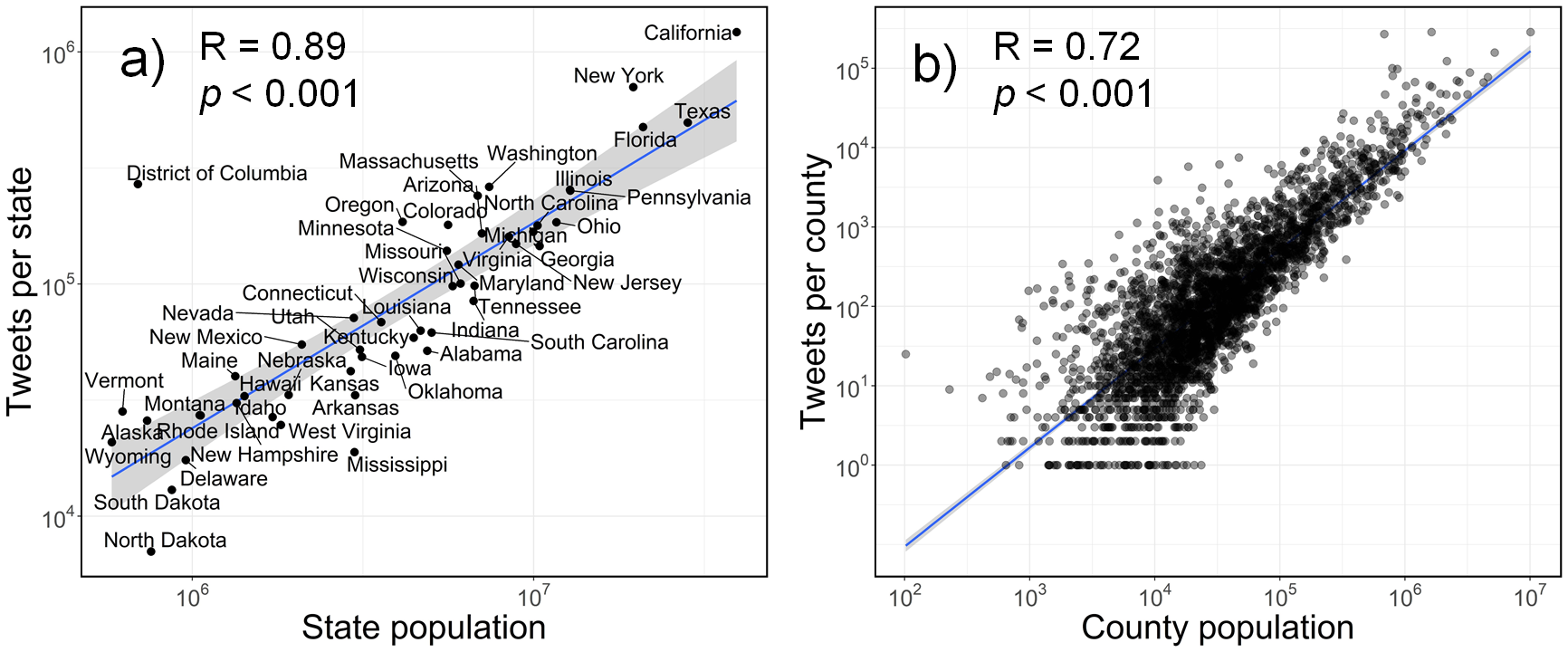


**Fig. S2.** Correlation plots of population and tweets volume used in our analyses at the **a)** state level and **b)** county level. The graphs were generated by R statistical computing software version 4.3.1 (https://cran.r-project.org/).

**Appendix (2): Model Validation**

*Validation using independent samples*

To evaluate the model’s performance (see methods for model implementation), we manually labeled an independent validation dataset. To ensure the validation dataset is balanced across the two categories and also is spatially representative, we randomly extracted 30 unique original tweets from each state. We labeled 1,500 tweets (736 ‘For’ and 764 ‘Against’) as validation samples. Our fine-tuned model achieved an overall accuracy of 0.91 and F1 score of 0.90. **Fig. S3** presents the error matrix of our prediction results.


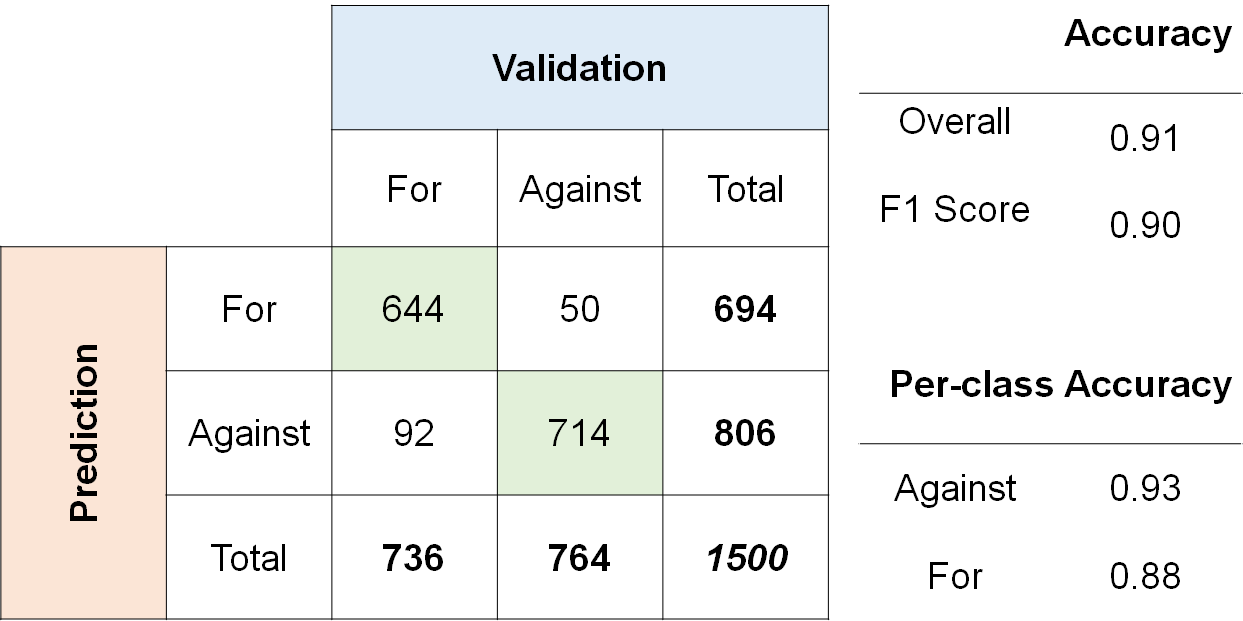


**Fig. S3.** Confusion matrix based on the 1,500 independent validation samples.

*Validation using external data*

We then compared our model predictions with US-wide estimates of climate change opinion from representative surveys. We used from three national survey projects that have tracked the dynamics of American climate change opinion over the past years. These projects are not fully consistent in the wording of survey questions, but they all measure nationwide percentages of agreement and disagreement on certain statements about climate change and can be used as proxies of believers and deniers.

The first dataset is from the Climate Change in the American Mind project (CCAM).^1^ The dataset^^[[1]](#footnote-1)^^ contains estimates of a wide range of national survey questions including climate change belief, risk perception, policy support and behavior. We used the estimates based on the “Do you think global warming is happening?” question.

The second dataset^^[[2]](#footnote-2)^^ is from the National Surveys on Energy and Environment (NSEE) project. The wording of the survey question is “is there solid evidence that the average temperature on earth has been getting warmer over the past four decades?”

The third dataset^^[[3]](#footnote-3)^^ is from the Climate Insight Survey project. The wording of the survey question is: “Do you believe earth temperature has probably been increasing over the past 100 years?” **Fig. S4** compares the estimated percentage of deniers as modeled by our study with those measured by national-level surveys. Surveys reveal that climate change deniers in the US range from 12% to 26% in the last five years. Results differ slightly between projects because of differences in sampling methods and survey design.


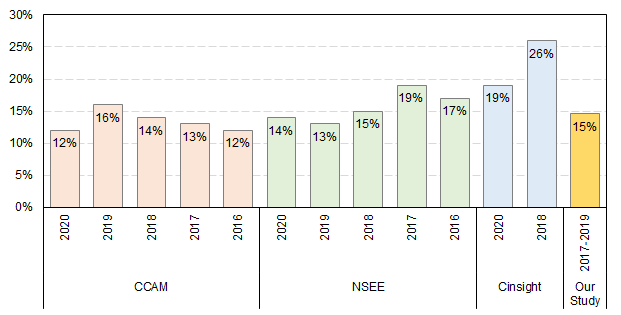


**Fig. S4.** **Estimated percentage of deniers from national representative surveys and our model**. CCAM represents Climate Change in the American Mind. NSEE represents the National Surveys on Energy and Environment. Cinsight represents the Climate Insight Survey. The graph was generated by R statistical computing software version 4.3.1 (https://cran.r-project.org/).

To validate our results at the sub-national level, we refer to the Climate Opinion Surveys developed by the Yale Program on Climate Change Communication. The state-level and county-level climate change opinion estimates from this project were produced using a downscaling statistical model based on national survey data. Validation results at the county level are illustrated in **Fig. S5** and specified in the main text.


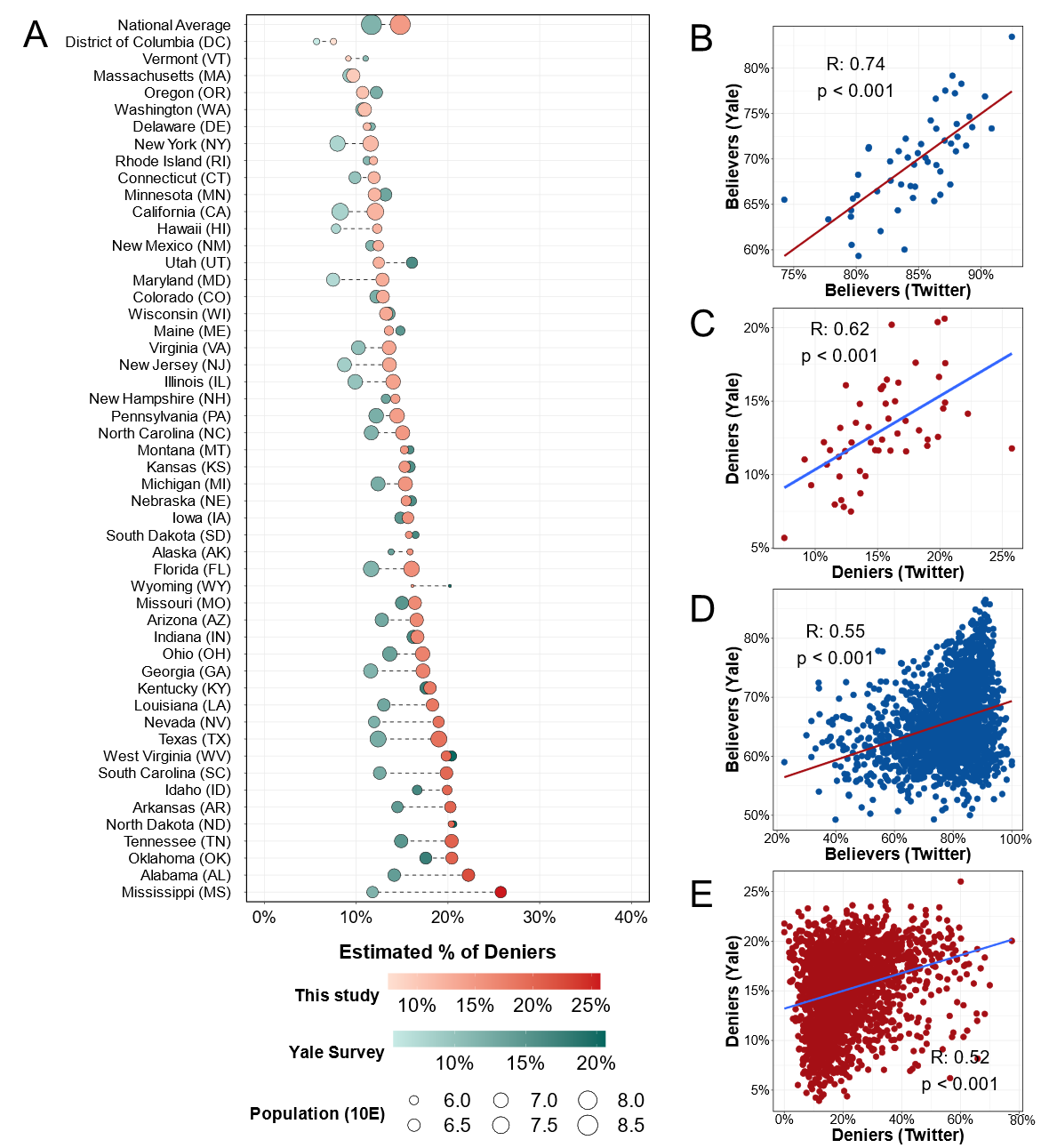


**Fig. S5. Comparing Twitter model results with Yale Climate Opinion Surveys.** (A) Comparison of percentages of deniers nationally and by state. Scatter plots (B) and (C) show the correlations of estimated percentages of believers and deniers between the Yale Survey and our predictions at the state level. Scatter plots (D) and (E) show the correlations of estimated percentages of believers and deniers between the Yale Survey and our predictions at the county level. The graphs were generated by R statistical computing software version 4.3.1 (https://cran.r-project.org/).

**Appendix (3): Additional Results**

*Detailed information for the top influencers*

By constructing the co-retweeted networks and calculating eigenvalue as a measure of influence for each user (see methods), we identify the top 30 influencers for deniers and believers, as presented in **Table S1** and **Table S2** respectively. The numbers of followers are updated in November 2020. Among the top 30 climate change deniers, we find 11 accounts ran by conservative news media or journalists who have a large number of followers highlighting their degree of influence. In addition, politicians, public personas and political commentators form another group of influential users. These people often have their own media channels and are actively engaged in producing climate denialism. Finally, we identify accounts that are individual bloggers or websites who have persistently spread dis/misinformation about climate change.

**Table S1.** Top 30 influencers in the community of climate change deniers as identified by our co-retweet network algorithms.

| **Rank** | **Followers** | **Detailed Information** |
| --- | --- | --- |
| 1 | 88,591,615 | Donald Trump. An American politician and businessman who served as the 45th President of the United States. |
| 2 | 20,166,050 | Fox News. An American multinational conservative cable news television channel based in New York City. |
| 3 | 285,667 | The Washington Examiner. An American conservative news website and weekly magazine based in Washington, D.C. |
| 4 | 827,141 | The Daily Caller. A right-wing news website based in Washington, D.C. |
| 5 | 2,748,739 | James Woods. An American actor and producer. He has been vocal in support of U.S. President Donald Trump. |
| 6 | 56,207 | Tony Heller. A blogger who have spread misinformation about climate change. His account has been suspended. |
| 7 | 38,665 | Friends of Science. A non-profit advocacy organization who rejects the established scientific consensus of climate change. |
| 8 | 25,826 | Climate Depot. A climate change denial website funded by Marc Morano who is a former Republican political aide. |
| 9 | 354,066 | Ryan Saavedra. A reporter at The Daily Wire. |
| 10 | 418,716 | The Washington Times. An American conservative daily newspaper published in Washington, D.C. |
| 11 | 3,500,753 | Ben Shapiro. An American conservative political commentator and media host who serves as editor emeritus for The Daily Wire. |
| 12 | 48,244 | Climate Realists. An organization who rejects science consensus about climate change. |
| 13 | 106,414 | Patrick Moore. A Canadian industry consultant and former activist who denies the climate change science. |
| 14 | 1,929,041 | Dinesh D'Souza. An Indian-American right-wing political commentator. |
| 15 | 668,644 | Chuck Woolery. An American former game show host and talk show host who is in favor of conservatism. |
| 16 | 1,928,752 | Charlie Kirk. An American conservative activist and radio talk show host who founded Turning Point USA. |
| 17 | 83,548 | Josh Kraushaar. A columnist at National Journal. |
| 18 | 6,200 | Michael Bastasch. A reporter for the conservative news site The Daily Caller. |
| 19 | 299,996 | Thebradfordfile. An alt-right social media-based twitter account. |
| 20 | 2,849,123 | Dan Bongino. An American conservative political commentator, radio show host, and author. |
| 21 | 337,647 | Kurt Schlichter. A senior columnist for Townhall.com |
| 22 | 29,468 | Watts Up With That. A blog promoting climate change denial that was created by Anthony Watts in 2006. |
| 23 | 109,266 | Alex Bruesewitz. The CEO of X Strategies LLC (a strategic consulting and media agency). |
| 24 | 1,062,673 | Sebastian Gorka. A Deputy Assistant to the President in the administration of U.S. President Donald Trump. |
| 25 | 173,110 | Kyle Hill. Former Co-Host and Producer of America Out Loud |
| 26 | 659,515 | Scott Adams. An American author and cartoonist. |
| 27 | 1,126,043 | Ryan Fournier. An American conservative activist and political commentator. Co-founder of the American youth group Students for Trump. |
| 28 | 1,206,712 | NEWS MAKER. A progressive social media-focused news organization. |
| 29 | 1,360,803 | Thomas Fitton. An American conservative activist and the president of Judicial Watch. |
| 30 | 1,506,016 | Sarah Palin. Former Governor of Alaska and GOP Vice Presidential Nominee |

Among the top 30 climate change believers, we find that members of the Democratic party are leading the narrative among believers. They are in favor of ambitious policy initiatives in addressing climate change. Popular mainstream media and journalists are also active and influential in reporting political trends and new scientific evidence regarding climate change. Several environmentalists, scientists, non-governmental organizations (NGOs) also make it in the list of top influencers.

**Table S2.** Top 30 influencers in the community of believers.

| **Rank** | **Followers** | **Detailed Information** |
| --- | --- | --- |
| 1 | 10,989,494 | Alexandria Ocasio-Cortez. An American Democrat politician. She has attempted to get legislation passed for a Green New Deal. |
| 2 | 4,059,721 | The Hill. An American newspaper and digital media company, focusing on politics, policy, business and international relations. |
| 3 | 11,061,179 | Bernard Sanders. An American Democrat politician and activist. He supports the ambitious Green New Deal to create jobs addressing climate change. |
| 4 | 51,520,630 | The Cable News Network. A news-based pay television channel headquartered in Atlanta, US ranked high in viewership. |
| 5 | 491,900 | Eric Holthaus. A meteorologist and climate journalist. |
| 6 | 48,469,027 | The New York Times. An American daily newspaper based in New York City with wide readerships. |
| 7 | 13,358,235 | Kamala Harris. An American Democrat politician. The 49^th^ and current vice president of the United States. |
| 8 | 1,772,936 | Daniel Rather. An American journalist and former national evening news anchor. |
| 9 | 2,734,020 | Adam Bennett Schiff. An American Democrat who serves as the U.S. representative for California’s 28^th^ Congressional District |
| 10 | 367,211 | Bill McKibben. An American environmentalist and journalist who has written on the impact of global warming. |
| 11 | 2,739,546 | NowThis News. A progressive social media-focused news organization. |
| 12 | 1,011,906 | Kyle Griffin. A senior producer of MSNBC. |
| 13 | 14,412,201 | Neil Tyson. An American science communicator. |
| 14 | 16,988,898 | The Washington Post. An American daily newspaper published in Washington, D.C. having a large national audience. |
| 15 | 5,385,804 | Elizabeth Warren. An American Democrat politician. As a candidate, she has detailed plans for climate change in 2020 presidential election. |
| 16 | 114,533 | Turnip2028 (The account has been deleted) |
| 17 | 30,074,777 | Hillary Clinton. An American Democrat Politician. She accepts the scientific consensus on climate change and supports climate policies like cap-and-trade. |
| 18 | 346,670 | Brian Schatz. An American Democrat Politician. Hawaii’s senior Senator. He has led key legislation on climate change. |
| 19 | 541,505 | The Climate Reality Project. A non-profit organization founded by Al Gore involved in education and advocacy related to climate change. |
| 20 | 164,766 | Michael E. Mann. American climatologist and geophysicist. He is the director of the Earth System Science Center at Pennsylvania State University. |
| 21 | 2,695,364 | Ilhan Omar. An American Democrat politician. One of the three principal organizers of the school strike for climate in the US. |
| 22 | 8,100,926 | The news division of the American broadcast television network NBC (National Broadcasting Company). |
| 23 | 79,013 | Peter Gleick. An American scientist with a focus on freshwater and human-caused climate change. |
| 24 | 434,027 | John Harwood. White House Correspondent for CNN. |
| 25 | 431,742 | Sheldon Whitehouse. U.S. Senator from Rhode Island, an American Democrat Politician. |
| 26 | 16,254,009 | American Broadcasting Company. An American multinational commercial broadcast television network headquartered in New York, NY. |
| 27 | 172,450 | Katharine Hayhoe. An atmospheric scientist and professor of political Scientist at Texas Tech University and the director of the Climate Science Center. |
| 28 | 341,815 | Natural Resources Defense Council. A US-based non-profit international environmental advocacy group. |
| 29 | 1,505,450 | Ted Lieu. An American Democrat politician |
| 30 | 1,159,127 | Robert Reich. An American economist and political commentator |

*Events that trigger tweet spikes*

We manually reviewed real-world events that potentially triggered these spikes and identified 17 events, as detailed below. These events include extreme weather events, new release of scientific reports, and political events related to climate change:

Table S3 Topic modeling results with date of event, the most frequently used keywords, hashtags and phrases, the volume of tweets and the stance of the users as classified by our model.

| **Date** | **Frequent words** | **Stance** | **Number of Tweets** | **Real-world events** |
| --- | --- | --- | --- | --- |
| 2017/10/23 | outspoken, fundamentalist, october, moore, rapaport, huffpostpol, roy, cking, presentations, govinslee, superevilbrian, dummy, rant, yanks | Denier | 395 | *Federal report says climate change costs billions*. A report released by the Government Accountability Office, a nonpartisan arm of Congress, warns that inaction on climate change has cost the country $350 billion over the last decade. The report urges President Trump to take immediate action to avoid these catastrophic costs. |
|  | cancels, yanks, e.p.a, abruptly, presentations, 1mtrees, madeinusa, teadorabeauty, bcorp, giveaway, muzzling, appearance, ep_actcampaign, equatorbanksact, blocks | Believer | 3368 |  |
| 2017/10/24 | 400, delingpole, 2017, papers, october, democrat's, congressional, donor, largest, relies, opens, halloween, failure, activism, nye | Denier | 633 |  |
|  | gao, costing, auditor, alphamundi, luxembourg, watchdog, cancels, abruptly, usgao, pioneering, taxpayers, olive, destem | Believer | 4730 |  |
| 2017/11/3 | deceptive, administration, gov't, slams, nov, critiques, defamation, koonin, traveling, november, clap, wsjopinion, steven, report | Denier | 523 | *Federal report says human causes climate change*. A federally mandated report, the Fourth National Climate Assessment (NCA4), confirms that climate change is real and human activities are the main cause of accelerating global warming. |
|  | wwf_act, 15,000, cpp, cop23, stillin, ward, 184, kilimanjaro, bonn, pitched, epascottpruitt, upped, harvey's, knit, capitols | Believer | 3340 |  |
| 2017/12/8 | allstars, bristol, encore, stomp, vites, dancing, airplane, moscow, parks, stocking, vomit, singing, featuring, shrinking, bay | Denier | 1484 | *South Texas Snowstorm*. Heavy Snowstorm hits South Texas and causes a wintry mix of rain and snow. The snowstorm lasted two days from the morning of December 7^th^ to the early morning of December 8^th^ 2017. |
|  | aniston, wrenching, iceless, crumbles, trumpthedestroyer, starving, snowing, melissa, destiny, gravity's, p.s.a, houstonsnow, removes, kimmel, snowintexas | Believer | 3434 |  |
| 2017/12/29 | hah, facing, cue, bundle, tweet, trolls, mocks, chelsea, year's, eve, politicususa, trillions, east, handler, bit | Denier | 2995 | *Trump tweets about cold weather and climate change*. President Trump tweeted that cities gripped by freezing temperatures on the East Coast could use some warming: “*In the East, it could be the COLDEST New Year’s Eve on record. Perhaps we could use a little bit of that good old Global Warming that our Country, but not other countries, was going to pay TRILLIONS OF DOLLARS to protect against. Bundle up!*” |
|  | searches, frigid, chilly, vinny, cue, snap, disprove, climatedenierinchief, gogolfing, refute, bitterly, disproves, battering, decides, extols | Believer | 7289 |  |
| 2018/01/02 | prophets, alternate, painting, sharks, individual, pink_about_it, chocolate, freezing, laughs, 40r, bombed, calgary, nationalism, trumpet, zoo | Denier | 992 | *North America Blizzard*. A powerful cyclonic blizzard that caused severe disruption along the East Coast of the United States in early January 2018. |
| 2018/01/10 | sues, 485, divests, scrubbed, websites, turtles, reuniting, 59, disbanded, ams2018, conocophillips, craigawelch, hawking, female, mudslides | Believer | 3588 | *NYC sues major oil companies over global warming.* The New York City government sued the world’s five largest publicly traded oil companies over climate change, including Shell and ExxonMobil. |
| 2018/04/22 | earthday, earthday2018, sinclair, credomobile, earthday18, perlberg, pbs, cleanpowerplant, balanced, 79, sundaymorning, 4.5m, happyearthday, networks, cbsnews | Believer | 3472 | Earth Day 2018 |
| 2018/08/02 | kavanaughscotus, confirmation, kavanaugh's, nathaniel, brett, losingearth, nytmag, credomobile, supreme, 1979, decade, surrounded, carculture, carrfire, jalopnik | Believer | 3412 | *Opposition to Kavanaugh’s Supreme court confirmation and California Wildfires*. A series of large wildfires erupted across California in mid-July to August 2018, which is one of the deadliest and most destructive wildfire seasons in the history causing 103 fatalities and $16.5 billion property damages. |
| 2018/09/12 | florence, gcas2018, climateresilience, assets, hurricaneflorence, stepup2018, gcas18, hurricanceflorence, runs, ceitdotorg, defied, electedofficial, burrowing, gcas, riseforclimate | Believer | 4579 | *Hurricane Florence.* A powerful hurricane that caused catastrophic damage in the Carolinas in September 2018 causing $24.23 billion in damage and 54 deaths. |
| 2018/10/08 | carries, u.n, ipcc, romer, nordhaus, nobel, dims, sr15, rosenstein, nobelprize, averting, awarded, 2040, ipcc_ch, prize | Believer | 4347 | *IPCC report warns rapid changes are needed.* The IPCC issued *The Special Report on Global Warming of 1.5°C*, warning that limiting global warming to 1.5°C would require rapid, far-reaching and unprecedented changes in all aspects of society. |
| 2018/11/27 | feds, snubs, conclusion, buried, assessment, ballistic, false, report, mikebastasch, hysterical, experts, blackfriday, moon, ocasio, cortez | Denier | 853 | *Trump administration claims Climate Report not based on facts.* President Trump casted doubt on a report by his own government warning of devastating effects from climate change and said “I don’t believe it”. |
|  | dismisses, mcnamee, bernard, rejectmcnamee, cybermonday, nca4, confirmations, buried, mythicals, ogres, pixies, bury, fairies, plsrt, connectthedots | Believer | 8807 |  |
| 2018/12/04 | schwarzenegger, terminate, riots, sanders, france, french, macron, bernie, forceful, leapfrogging, wishes, macron's, stakes, hall, detail | Denier | 1224 | *UNFCCC COP 24.* The 2018 United Nations Climate Change Conference (COP24) was held between 2 and 15 December 2018 in Katowice, Poland. |
|  | 24hoursofreality, purposing, cop24, cove.tool, climatetownhall, ocasio2018, attenborough, fishermen, realism, poland, greennewdeal, ocasio, bernie, cortez, g20 | Believer | 5686 |  |
| 2018/12/10 | kuwait, arabia, saudi, cop24, hopefuls, poland, agu18, dueling, resists, fugu, delicacy, mutant, balk, agendas, greennewdeal | Believer | 4352 | *US rejects affirming global warming’s severity.* The US government rejected language strongly affirming the severity of global warming at the COP24. |
| 2019/04/22 | earthday, earthday2019, waikiki, quiz, parents, 99.9999, happyearthday, martyrs, gruel, peters4michigan, hawaii's, 207, queensswab, greennewdeal, ipsos | Believer | 8488 | Earth Day 2019 |
| 2019/04/30 | beto, o'rourke, popcorn, coveringclimatenow, apples, ceres19, zuckerberg, beto's, o'rourke's, altering, kimseverson, betoorourke, getusthere, aoc, foods | Believer | 4378 | *O’Rourke releases $5 trillion climate change proposal.* Beto O’Rourke, who sought the 2020 Democratic nomination for President, announced a $5 trillion plan to fight climate change. |
| 2019/05/14 | aoc, biden, nye, rant, filled, bomb, psycho, gatewaypundit, ocasio, cortez, bans, tyranny, junk, apple | Denier | 1035 | *Bill Nye curses out climate deniers.* Bill Nye, a science communicator and television presenter, cursed out climate deniers in the latest call to action. |
|  | biden, nye, nye's, 415, profanity, ocasio, oliver's, oliver, cortez, aoc, ppm, biden's, fiery, bombs, billnye | Believer | 4748 |  |

*Summarizing major topics for deniers and believers*

To understand how each group perceives real-word events, we implemented the Latent Dirichlet Allocation (LDA) model, which is a machine learning technique, to extract topics from original tweets of deniers and believers respectively. To specify the number of topics when running the LDA topic modeling since it is an unsupervised algorithm, we referred to a five-category classification scheme following Cook’s (2019)[^4^](#_heading=h.1t3h5sf) categories of misinformation, namely, a) climate change is/is-not real (*Reality*); b) humans are/are-not the main cause (*Cause*); c) the impacts are/are-not serious (*Impact*); d) the experts are reliable/unreliable (*Expert*); e) there is still time to adapt/solutions offered are inefficient (*Solution*). We trained the model with different numbers of topics and compared their coherence values as a measure to judge the modeling quality (**Fig. S6**). The LDA model will give corresponding keywords for each topic (see examples in Table S3). We manually reviewed these keywords and found they aligned well with the five-category scheme. For the “against” tweets, however, we did not find keywords related to the topic of *Impact*. We thus only kept the four topic categories (excluding *Impact*) for the “against” tweets.


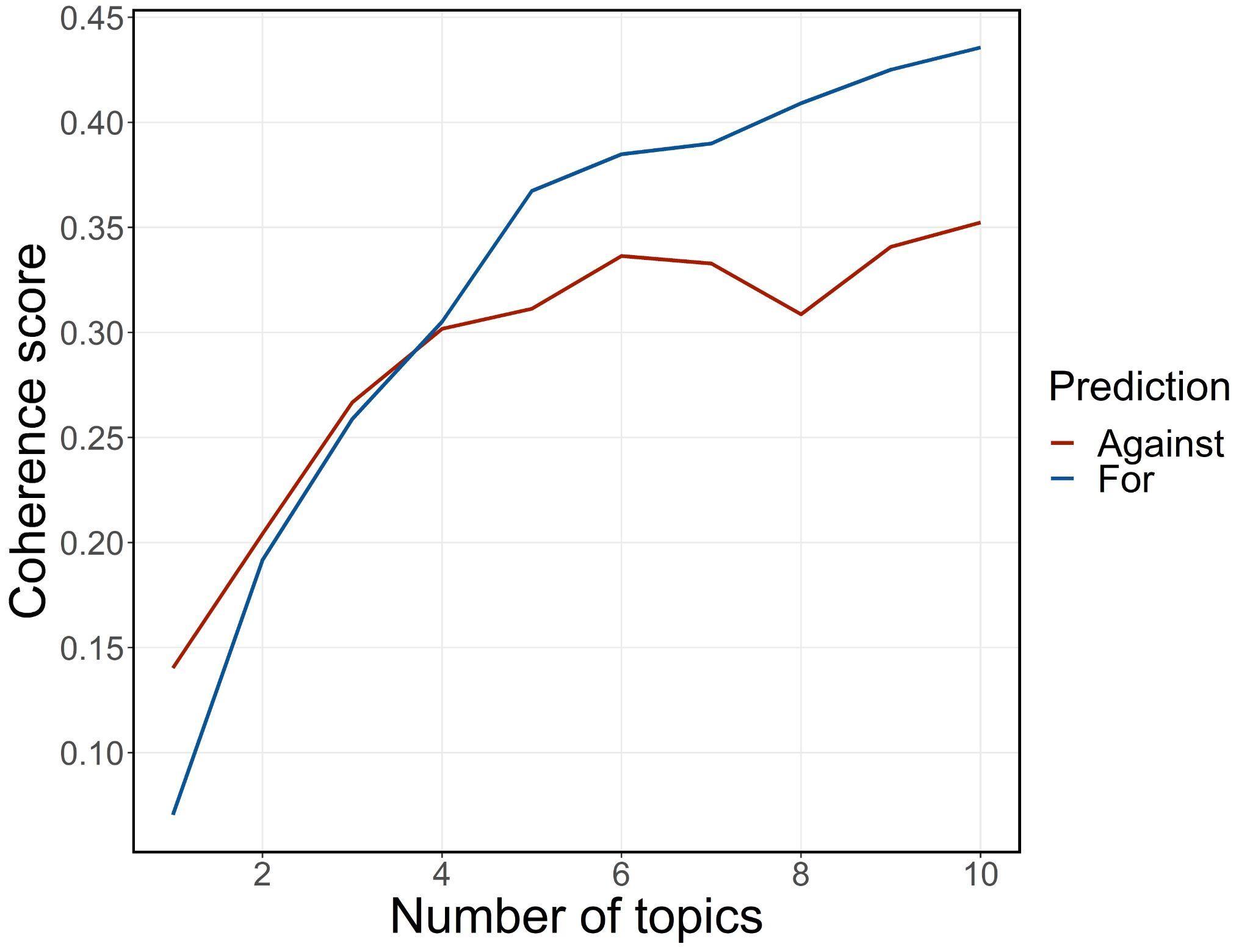


**Fig. S6.** Coherence scores by number of topics. The graph was generated by R statistical computing software version 4.3.1 (https://cran.r-project.org/).

*Explanations for each topic and examples tweets*

Here we give brief explanations for each topic that believers and deniers have engaged in and provide some typical tweets as examples.^5^

*1. Reality*. Tweets related to this topic concentrate on whether climate change is really happening. Tweets of this topic are mostly posted during extreme weather events. For example, deniers tweet when an extreme cold weather event occurred and the tweet content questions the reality of global warming. In contrast, believers are attributing these weather events to climate change.

- *In the East, it could be the COLDEST New Year's Eve on record. Perhaps we could use a little bit of that good old Global Warming that our Country, but not other countries, was going to pay TRILLIONS OF DOLLARS to protect against. Bundle up! (Deniers)*
- *He doesn't do all the things he wants the US to do. Global warming is the perfect power grab. Woodrow Wilson tried it out- people were closer to nature then- it didn't get far. The science HF talks about is the science individuals have made up. (Deniers)*
- *California experienced its hottest single month in 124 years of recordkeeping. For the contiguous U.S. as a whole, it was the 11th-hottest July on record, with almost every state coming in warmer than average. #climatechange (Believers)*

*2. Cause.* People who tweet about this topic are debating on if human activities are responsible for accelerated global warming. Deniers claim that climate has been changing naturally and at a geological time scale and human activities do not have any influence on this. Believers on the other hand emphasize the role of human activities and fossil fuels and GHG emissions on rising temperatures and climate change.

- *It's time to remove the UNFCCC. We don't need a group that tells us there is climate change, when it is a natural thing that occurs each season. And temps haven't changed for eons. Hottest temps: 1100 AD-1400 AD. Temps change +or-, about every 300yrs, then go back to normal. (Deniers)*
- *Burning fossil fuels is not one of these idealized circumstances; greenhouse gas emissions represent a market failure (negative externality) because the costs of climate change are distributed across society (socialized) while the benefits of obtaining the energy are privatized. (Believers)*

*3. Impact.* Tweets with this topic are mainly about the broad impacts of climate change. Interestingly, tweets related to this topic are heavily posted by believers:

- *Such a great example of environmental restoration, including vital flood projects. Efforts like the Shoreline Project will only become more important as climate change causes sea levels to rise and threaten our communities. (Believers)*

*4. Expert.* Tweets engaged in this topic are discussing if climate change is a scientific consensus or if climate scientists are reliable. Believers are advocating for the scientific consensus that human-caused climate change is scientific consensus and most experts agree. Deniers show strong opposition to the scientific consensus of climate change and firmly view climate science as a wholesale fraud.

- *Climate Change scientists who faked data are now desperately archiving it to cover their tracks (Deniers)*
- *Maybe you should start here with some basic stuff. Then, if you truly want to understand the science and not regurgitate tired old claims, you will have no trouble finding credible science that shows how humans are warming the planet. Best regards. (Believers)*

*5. Solution.* Tweets of this topic mainly communicate if we should take actions to tackle climate change. Believers emphasize the importance to take measures to mitigate emissions and adapt to climate change. Especially during the Earth Day and IPCC conferences, tweets with this topic are mainly about information campaigns related to climate change. Also, some tweets are expressing anger over Republicans who view climate change as a low-priority issue, and criticizing President Trump’s and his administration’s inaction against climate change. However, deniers regard climate change as an item of the political agenda from Democrats for wealth redistribution. These tweets strongly oppose all types of climate policies, such as renewable energy policies and fossil fuel taxation.

- *The effects of climate change can be irreversible. We must act now! #ActOnClimate #environment (Believers)*
- *We will potentially have a climate change speaker on Earth Day! But we need a little help with funding! Please help us educate the campus on such an important topic and donate here (Believers)*
- *It's real shame that you support a TERRIBLE Presidentwho thinks climate change is a hoax, guts the EPA; laws that are trying to save this planet before the oceans go stagnant, the forest all burn; most animals go extinct WORST PRESIDENT EVER (Believers)*
- *Because of the save the planet/global cooling ('70's) warming ('80's) climate change ('90's-now) political agenda of wealth redistribution. Started when all the folks got tired of how frigged up Cali was and then they all moved to Seattle. Then started moaning about Spotted Owls. (Deniers)*

**Table S3.** Major topics and keywords detected from the Latent Dirichlet Allocation (LDA) model based on tweets of deniers and believers.

| **Topic** | **Keywords** |
| --- | --- |
| Reality | Deniers: cold, day, snow, hot, today, winter, real, degree, hoax, money, trump, liber, fake, world, al gore, tax, big, scam  Believers: year, day, weather, California, extreme, high, bad, due, time, fire |
| Cause | Deniers: year, earth, time, man, human, planet, natural, fire, god  Believers: energy, state, emission, power, carbon, oil, plan, clean, pollution |
| Impact | Believers: impact, water, city, effect, communicate, research, study, risk, natural, food |
| Expert | Deniers: scientist, fact, real, data, theory, wrong, evidence, true, scientific  Believers: science, real, human, scientist, planet, earth, life, fact, thing, denier |
| Solution | Deniers: agenda, democrat, problem, government, control, world, issue, Obama  Believers: issue, policy, health, president, American, care, support, political, administration |

**SI References**

1 Littman, J. & Wrubel, L. Climate Change Tweets Ids. *Harvard Dataverse*, (2019).

2 *Hydrator*, <<https://github.com/docnow/hydrator>> (2020).

3 OpenStreetMap. <<https://www.openstreetmap.org/> > (2020).

4 Cook, J. in *Handbook of research on deception, fake news, and misinformation online.* *Advances in media, entertainment, and the arts (AMEA) book series.* 281-306 (Information Science Reference/IGI Global, 2019).

1. Data available at <https://climatecommunication.yale.edu/visualizations-data/ycom-us/> [↑](#footnote-ref-1)
2. Data available at <https://www.muhlenberg.edu/aboutus/polling/surveys/national/energyandenvironmentsurveys/> [↑](#footnote-ref-2)
3. Data available at <https://www.rff.org/publications/data-tools/climate-insights/> [↑](#footnote-ref-3)
